# Advanced 3D Printing of Polyetherketoneketone Hydroxyapatite Composites via Fused Filament Fabrication with Increased Interlayer Connection

**DOI:** 10.3390/ma17133161

**Published:** 2024-06-27

**Authors:** Krzysztof Rodzeń, Eiméar O’Donnell, Frances Hasson, Alistair McIlhagger, Brian J. Meenan, Jawad Ullah, Beata Strachota, Adam Strachota, Sean Duffy, Adrian Boyd

**Affiliations:** 1 School of Engineering, Ulster University, York St, Belfast BT15 1ED, UK; odonnell-e19@ulster.ac.uk (E.O.); hasson-f3@ulster.ac.uk (F.H.); a.mcilhagger@ulster.ac.uk (A.M.); bj.meenan@ulster.ac.uk (B.J.M.); j.ullah@ulster.ac.uk (J.U.); duffy-s36@ulster.ac.uk (S.D.); 2 Institute of Macromolecular Chemistry v.v.i., Academy of Sciences of the Czech Republic, Heyrovskeho nam. 2, 162 00 Praha, Czech Republic; beata@imc.cas.cz (B.S.); strachota@imc.cas.cz (A.S.)

**Keywords:** additive manufacturing, crystallization kinetics, advanced semicrystalline polymers, 3D printing, polyetherketoneketone, hydroxyapatite

## Abstract

Additively manufactured implants, surgical guides, and medical devices that would have direct contact with the human body require predictable behaviour when stress is applied during their standard operation. Products built with Fused Filament Fabrication (FFF) possess orthotropic characteristics, thus, it is necessary to determine the properties that can be achieved in the XY- and Z-directions of printing. A concentration of 10 wt% of hydroxyapatite (HA) in polyetherketoneketone (PEKK) matrix was selected as the most promising biomaterial supporting cell attachment for medical applications and was characterized with an Ultimate Tensile Strength (UTS) of 78.3 MPa and 43.9 MPa in the XY- and Z-directions of 3D printing, respectively. The effect of the filler on the crystallization kinetics, which is a key parameter for the selection of semicrystalline materials suitable for 3D printing, was explained. This work clearly shows that only in situ crystallization provides the ability to build parts with a more thermodynamically stable primary form of crystallites.

## 1. Introduction

Additive manufacturing (AM), commonly referred to as three-dimensional (3D) printing, has revolutionized the design and manufacturing industries over the past decade [1]. This technology enables the production of complex, customized, and intricate parts through a layer-by-layer approach, eliminating the need for additional processing [2]. Unlike traditional subtractive manufacturing methods, AM builds parts from the ground up, allowing for a greater design flexibility and efficiency. This technology encompasses various techniques such as stereolithography, selective laser sintering, and the widely used fused deposition modelling, all of which follow the fundamental principle of stacking successive layers to create parts. Among the various AM technologies, fused deposition modelling is favoured for manufacturing medical devices due to its straightforward fabrication process, cost-effectiveness, extensive material customization options, and capability to produce intricate components [3,4].

The rapid advancements in AM have facilitated its widespread adoption across diverse sectors, including automotive, aerospace, and biomedical, significantly transforming these industries [5]. Additive manufacturing (AM) offers significant benefits in the medical field, particularly in the fabrication of complex and personalized medical devices, pharmaceuticals, and surgical tools with an enhanced resolution, precision, reliability, and consistency. The use of AM to create custom implants, fixtures, and surgical tools has not only reduced surgical and recovery time, but also improved procedural outcomes [6,7,8].

One of the most studied materials from the PAEK (polyaryletherketone) family of polymers is PEEK (polyetheretherketone), which reveals exceptional mechanical properties after injection moulding [9]. However, the additive manufacturing (AM) process for this semicrystalline polymer proved challenging, resulting in suboptimal mechanical properties for 3D-printed parts. This challenge is particularly pronounced in the Z-direction, perpendicular to the deposited printed layer, due to the polymer’s rapid crystallization rate. When PEEK was 3D-printed in the XY-direction, its tensile stress was recorded between 49 and 89 MPa [10,11,12]. There are limited number of studies which refer to measuring the interlayer connection, which is crucial, as this is the weakest point of FFF 3D-printed parts. When a PEEK/CF composite was printed, a tensile strength of 36.3 MPa was achieved in the Z-direction [13]. In the case of PEEK blends, it was possible to achieve a tensile strength of up to 25 MPa [14]. This clearly indicated a poor interlayer connection with a tensile performance between 3.9 and 20 MPa, respectively, depending on the PEEK grade [15,16].

Our prior investigations into PEEK/HA composites revealed that 3D-printed components in the XY printing direction can achieve an Ultimate Tensile Stress (UTS) in the range of 79.5–94.2 MPa. This range closely approximates the performance exhibited by human femoral cortical bone, which typically ranges from 71 to 97 MPa [17]. However, the mechanical properties in the Z-direction for the specimens utilized in this research could not be measured. This was due to the extensive delamination of the 3D-printed part during the waterjet extraction of the tensile and DMA specimens, thereby revealing the weak mechanical performance of the printed PEEK material.

The advanced semicrystalline commercial market is not broad, and besides fast-crystallizing PEEK, it is possible to source PEKK (polyetherketoneketone) in three variations. PEKK 8000 has comparable crystallization kinetics to PEEK, PEKK 7000 has moderate kinetics, and PEKK 6000 has slow crystallization kinetics [18,19,20]. PEEK is the most studied material from the advanced PAEK thermoplastic family. It has been utilized to develop PEEK/HA scaffolds, aiming to enhance cell attachment and mineralization [21]. Alternatively, bioglass can replace HA to enhance bioactivity and bone integration during the filament-manufacturing process [22,23], followed by PEEK 3D printing parameters’ optimization [24].

In this study, PEKK 6002 (the last number in the name indicates its moderate viscosity) was utilized due to its slower crystallization rate, making it the most promising material for medical applications. Its main disadvantage is the impracticability of 3D printing this material crystallized in situ; building the crystal fraction post-processing at temperatures between the glass and melting temperature is necessary. This process leads to secondary crystallites forming with a lower mechanical and thermal performance [25,26,27]. Nevertheless, these limitations do not undermine the material’s suitability for the production of ‘made-to-measure’ medical implants or surgical guides using AM. The material printed as an amorphous body still resists the high temperature during sterilization within an autoclave, and its glass transition temperature is 160 °C. Additionally, it possesses a better impact resistance and higher strain under load, which is especially important when HA composites are prepared [28,29]. In previous work, in order to obtain osteoblast cell morphology with spread-out elongated filopodia for PEEK/HA, it was necessary to incorporate at least 10–20 wt% of HA [30]. Such a large amount of the ceramic filler certainly improved the tensile modulus and ultimate tensile stress, but the tensile strain remained below 3%. This was due to the high ceramic filler content connected with an elevated level of crystallinity. For these reasons, the PEKK 6002 grade was the most promising candidate for making ‘made-to-measure’ implants due to its amorphous character. PEKK can find application in the medical field, having already obtained approval from the Food and Drug Administration for use in oro-maxillofacial and spinal surgeries [31]. However, PEKK is bioinert and can lead to weak osseointegration, limiting the formation of tissue around the implant. To address this issue and improve cell attachment, bioactive calcium phosphate, specifically hydroxyapatite (HA), was used in this study. The availability of HA on the surface supports the appropriate osseointegration [32]. Furthermore, it has been found that nanostructured orthopaedic implants can reduce the risk of infection [33]. Recent studies provided the opportunity to build confidence in successfully determining the performance of specimens printed in the Z-direction when PEKK 6002 is used [34]. The Z-direction is the weakest point of the 3D-printed body, and its performance reflects the mechanical performance of the whole potential orthotropic implant.

**Figure 1:**
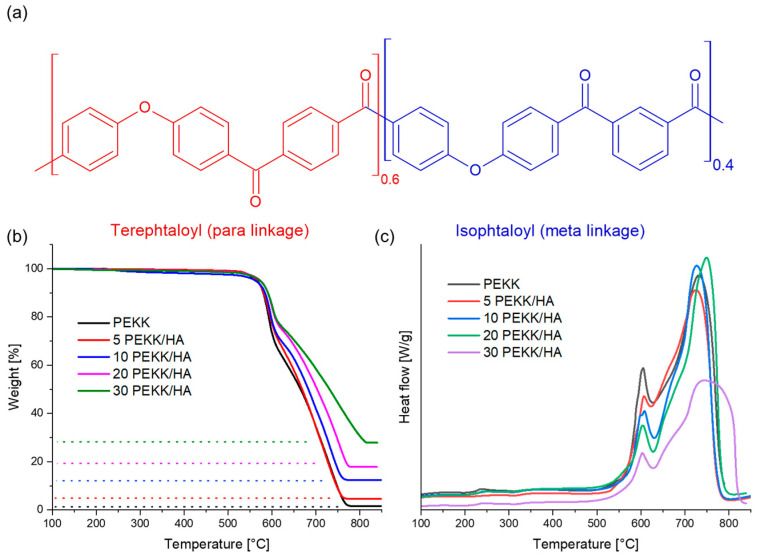
(**a**) Chemical structure of PEKK 6002 and thermogravimetry results for the PEKK/HA composite filaments and pure PEKK. The heating rate used was 20 °C/min, with (**b**) weight % plotted against temperature on the left side and (**c**) heat flow versus temperature on the right side. The dotted lines correspond to the inorganic residue of hydroxyapatite after the composite undergoes oxidative decomposition.

Diphenyl ether is used in the synthesis process, reacting with terephthalic acid to form terephthaloyl para linkages or with isophthalic acid to form isophthaloyl meta linkages. The regularity of the polymer chain decreases with higher concentrations of isophthalic acid, resulting in slower crystallization kinetics (see Figure 1a).

The objective of this study was to explore the mechanical characteristics of PEKK and PEKK/HA composites, varying the HA content from 5 to 30 wt%. The printing configurations included both the XY plane and the Z-direction. Changes in the filler concentration can influence the crystallization kinetics, which, in turn, affect the thermal and mechanical properties of the printed composites. An in-depth analysis of the crystalline structure formed during in situ 3D printing is crucial to determine the predominant crystal form, i.e., whether it is the thermally stable primary form or the less stable secondary form. Another aspect of this research is to evaluate the accessibility of the filler for cell attachment on the surface of the 3D-printed parts, considering the HA distribution and the tendency for agglomeration, which are essential factors in providing a successful material for medical purposes. According to the best of our knowledge, we were not able to find information about PEKK/HA composites used in additive manufacturing. Additionally, research related to the filler’s influence on the kinetics of advanced semicrystalline materials and its further impact on the 3D printing process and material properties was not conducted. This article emphasizes the significance of studying the kinetics of semicrystalline materials and evaluating their performance in the Z printing direction within the additive manufacturing processes.

## 2. Experimental

### 2.1. Materials

Unsintered HA powder, known as CAPITAL^®^ R (Plasma Biotal Ltd., Buxton, UK), was blended with PEKK 6002 powder (Hebei Bonster Technology Co., Ltd., Shijiazhuang, China). Utilizing a twin-screw extruder (Rheomex PTW16/40 OS, HAAKE, Vreden, Germany) with a 40 mm length and 16 mm diameter, continuous filaments with varying HA filler contents (0, 5, 10, 20, and 30 wt%) were produced.

The HA/PEKK filaments, with a diameter of 1.75 (±0.10) mm, were extruded with a screw speed of 45 rpm and die temperature of 340 °C. The extruder featured specific screw elements, including feed elements for material conveying, mixing elements with 0°, 30°, 60°, and 90° in the melting and mixing section, and additional elements for functions like feeding, conveying, reverse conveying, and venting. To maintain a consistent filament diameter between batches as the filler content increased, the winding speed and feed rate were adjusted to accommodate the increased viscosity of the melt.

Before extrusion, the powders underwent mixing, stirring, and drying for 24 h at 150 °C in an oven with air circulation.

The printing process was performed on a modified Ultimaker 2+ (Ultimaker, Geldermalsen, The Netherlands). The printing parameters remained consistent for both the PEKK 6002 matrix and its composites. The printer and slicer settings were analogic to our previous work, where it was determined that a current of 2.5 A on the two 500 W lamps was determined as optimal in order to print stable and undeformed parts [22].

### 2.2. Methods

X-ray diffraction (XRD) measurements were conducted, employing an Empyrean diffractometer (Malvern Panalytical, Almelo, The Netherlands) at 45 kV and 40 mA, utilizing Cu Kα radiation (*λ* = 1.54187 Å). Diffractograms were acquired within the 2-Theta (θ) range from 3° to 70°, with an angular step interval of 0.0394°. The XRD plot served for both crystallinity determination, calculated as the area of the crystalline peaks divided by total area of peaks multiplied by 100, and crystal grain size estimation using the Scherrer equation *τ* = (*K*∙*λ*)/(*β*∙*cosθ*), where *τ* represents the mean grain size, *K* is a shape factor (equal to 1), *λ* is the X-ray wavelength, *β* is the full width at half maximum (FWHM), and *cosθ* is the Bragg angle.

A scanning electron microscopy Energy Dispersive X-ray, (SEM/EDX) analysis was carried out employing a Hitachi SU5000 (Oxford Instruments, Abingdon, UK) field emission instrument, which was equipped with an associated X-MaxN 80 mm^2^ silicon drift detector. An acceleration voltage of 10 KeV was applied during imaging. To enhance the conductivity of the samples, their surfaces were coated with Au/Pd at a 60/40 ratio.

Dynamic Thermomechanical Analysis (DMTA) was conducted on the printed specimens using rectangular specimens, which were obtained after waterjet cutting with dimensions of 50 × 10 × 3 mm. The analysis was performed with an ARES G2 rheometer (TA Instruments, New Castle, DE, USA). The temperature dependencies for the storage shear modulus (*G*′), the loss factor (*G*″), and (*Tan*(*δ*)) ranging from 50 to 380 °C with a heating rate of 10 °C/min were followed.

Thermal imaging was conducted using a GTC 400 C thermal camera from Bosch, Germany, capable of detecting temperatures within the range from −10 °C to +400 °C. GTC Transfer Software 1.7.1.0 was used for the analysis of the captured images.

The tensile properties were determined using the Instron 5500R Model (Instron, Norwood, MA, USA) at room temperature. The 3D-printed specimens were produced according to the ASTM 638 Type IV and V. For Type IV with an overall length of 115 mm, length of narrow section of 33 mm, overall width of 19 mm, gage length of 25 mm, radius of fillet of 14 mm, and Type V with an overall length of 63.5 mm, length of narrow section of 9.53 mm, overall width of 9.53 mm, gage length of 7.62 mm, radius of fillet of 12.7 mm, 5 specimens were manufactured and tested with a testing speed of 5 mm/min.

Differential Scanning Calorimetry (DSC) analyses were conducted using a DSC 25 instrument (TA Instruments, New Castle, DE, USA) equipped with an autosampler and calibrated with pure indium. A 5 mg sample was extracted from the centre of a rectangular specimen measuring 50 mm in length, 10 mm in width, and 3 mm in thickness. The analysis involved a cooling/heating cycle with a heating rate of 10 °C/min within the temperature range from 50 to 420 °C. Additionally, an isothermal study was undertaken between 180 and 300 °C with 10 °C increments for a duration of 2 h.

Thermal Gravimetric Analysis (TGA): mass loss vs. temperature and heat flow vs. temperature curves were acquired using a TA SDT Q600D thermogravimetric analyzer (TA Instruments, New Castle, DE, USA). The mass of the samples that underwent measurement was 10 mg at a heating rate of 20 °C/min and air was used as the purge gas within the temperature range from 50 to 850 °C.

Waterjet cutting was performed on Wazer (WAZER, Yonkers, NY, USA). The wall specimen was attached to the base with 4× M5 button head socket caps to keep the specimen stable during the cutting process at a speed of 0.2 cm/s. DXF files were produced via Fusion 360 (Autodesk, San Francisco, CA, USA) following ASTM 638 Type V.

## 3. Results and Discussion

### 3.1. Thermal Stability

The successful blending of PEKK/HA composite filaments with a filler content ranging from 0 to 30 wt% was verified through thermogravimetric analysis (TGA), as illustrated in Figure 1b. The residual inorganic content post-oxidative treatment in the performed analysis corresponded closely to the quantity of HA combined with PEKK during the extrusion batch preparation (refer to Figure 1b). The oxidative decomposition of both PEKK and the composite material initiated above 525 °C, reaching its peak at 600 °C. The height of this peak diminished with an increase in the filler content, as the filler took the place of the organic matrix, which was susceptible to oxidative degradation. The identified exothermic peak, which extended from 525 °C to 625 °C, refers to the breaking of bonds within ethers and ketones as a result of oxidation, leading to subsequent carbonization. The resultant by-products of carbonization experienced additional oxidation, giving rise to a second exothermic peak at approximately 700 °C (see Figure 1c). It is important to highlight that, in the context of 30% PEKK/HA, there was a noticeable decrease in the peak height along with broadening. This observation implied a modification in the activation energy associated with this process, a phenomenon previously documented in studies involving hydroxyapatite composites within other polymer matrices [35].

### 3.2. Thermal and Mechanical Behaviour

The phenomenon of filler acting as a nucleation agent is well-known. However, the isothermal study revealed that this effect was observed only for a low content of filler in the composite material. A similar finding can be noted for semicrystalline composites with commodity and engineering plastics, where it was found that the isothermal crystallization rate increased by up to 7.5 wt% in PP/SiO_2_ and PA12/Cu composites [36,37].

**Figure 2:**
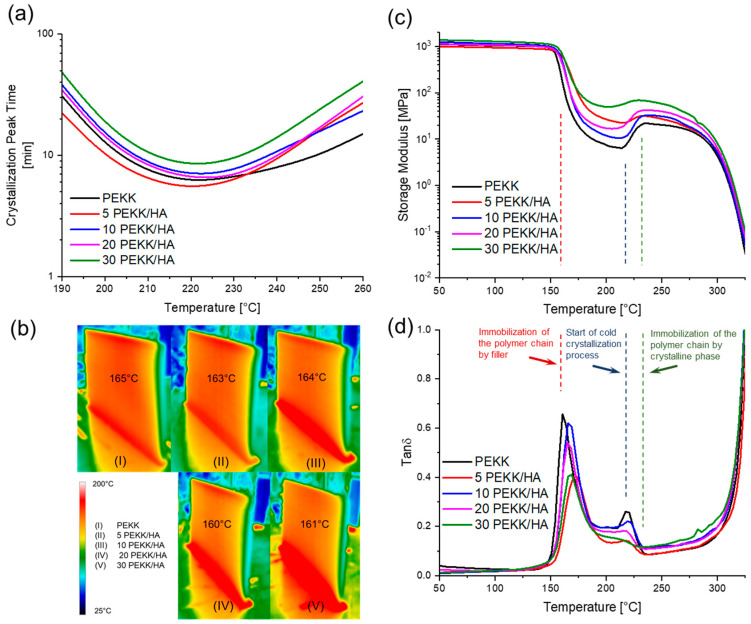
Thermal and mechanical study of PEKK and PEKK/HA composites includes the following: (**a**) crystallization kinetics, measured by following the DSC isothermal crystallization peak time, studied between 180 °C and 260 °C, (**b**) thermal images captured after the completion of 120 × 70 × 3 mm thick specimens’ 3D printing for both PEKK and PEKK/HA composites. All samples were printed under the same conditions, with the current set to two 500 W units operating at 2.5 A, (**c**) DMA profiles obtained with a heating rate of 10 °C/min, including the storage modulus, and (**d**) DMA profiles obtained with a heating rate of 10 °C/min, including *Tan δ*.

To evaluate the effect of the filler on the crystallization rate, a DSC isothermal study was performed between 180 °C and 260 °C with an increment of 10 °C. Initially, each sample was preheated to 400 °C and maintained for 5 min to remove its thermal history. It was then rapidly cooled down to the desired temperature, at which isothermal conditions were applied for one hour. From the obtained heat flow vs. time plots, the time of the peak at different temperatures was estimated for each composition in the isothermal study (see Supplementary Figure S1). The data obtained were used to create a plot of the crystallization peak time versus temperature (see Figure 2a).

The DSC study showed that the highest crystallization rate was observed for the 5 PEKK/HA composite, where the filler increased the crystallization rate at 220 °C to 5.3 s, compared to the PEKK matrix with 6.3 s at the same temperature. Higher concentrations of HA filler led to a reduction in the crystallization rate at 220 °C, reaching 8.6 s for the highest concentration, where 30 wt% of the filler was incorporated into the polymer matrix.

This is extremely important and clearly illustrates the different behaviour of the composite materials during the DMA analysis. Three typical regions can be distinguished in the DMA thermograms. The first begins at a temperature around 160 °C, associated with the glass transition temperature (*T_g_*), which increases with higher filler loading up to 167 °C (see *Tan δ* peak position in Figure 2d). A characteristic drop in mechanical properties is observed, indicating viscoelastic behaviour (see Figure 2c,d, red dashed line). The second region begins at the modulus minimum around 215 °C (blue dashed line), followed by a step increase in the storage modulus with a local maximum around 235 °C for all specimens (green dashed line), which is typical for PEKK [38]. This aligns well with the crystallization peak time, where materials reach the temperature of the fastest crystallization rate, and the rise in the storage modulus is associated with the generation of the crystalline phase during the so-called cold crystallization process. Below that temperature, crystallization is limited by slow diffusion processes (Figure 2b). When temperatures in the DMA chamber were close to the temperature at which the crystallization rate reached its maximum, the cold crystallization process was observed, associated with an increase in the storage modulus. The *Tan δ* peak at 215 °C corresponded to the released mobility of the polymer chains and was present at the same temperature where the storage modulus reached its local minimum. The second *Tan δ* peak reached its local minimum at 235 °C, which was associated with the storage modulus local maximum at the same temperature and with the fact that the crystallization kinetics slowed down due to nucleation.

After the gradual incorporation of the filler, with a modulus around 80–120 GPa, it is possible to expect a rising trend in the storage modulus with a gradual increase in the filler amount and a decrease in the *Tan δ* peak due to the immobilization of the polymer chains by the filler [39]. Such a tendency could be observed for PEKK, 10 PEKK/HA, 20 PEKK/HA, and 30 PEKK/HA, but it was not observed when the 5 PEKK/HA composite was used. The small difference in crystallization kinetics caused an unusually strong effect on the mechanical properties of the 3D-printed sample, suggesting that the activation energy for nucleation and crystal growth was reduced at a low concentration of filler, which was already observed for semicrystalline polymers [40].

The reported Tensile Modulus, Ultimate Tensile Strength (UTS) in [MPa] unit, and Tensile Strain in (%) are presented as the mean value ± standard deviation (with *n* = 5). The crystallinity fraction (*X_c_^XRD^*) was determined by calculating the integral of the crystalline phase divided by the sum of integrals of the amorphous and crystalline phases. The Average XRD grain size (in nm) was computed from the Full Width at Half Maximum (FWHM) peak around 2θ 18.6°. The tensile Modulus and Ultimate Tensile Strength values are reported as the mean value ± standard deviation (*n* = 5).

Faster crystallization kinetics led to the formation of a better-defined crystalline phase, which was confirmed after analysing the XRD data. The crystallinity was calculated for the specimens printed in the XY-orientation. For PEKK, the total crystallinity was at the level of 13.1% and increased to 14.4% for 5% PEKK/HA, then dropped down to reach 8.3% with 30 PEKK/HA (see Table 1). These data align with the crystallization kinetics (see Figure 2a). For the samples printed under the same conditions, the fastest crystallizing 5 PEKK/HA reached the highest level, while it was the lowest for PEKK and 30 PEKK/HA, showing the same tendency. By the maintaining the conditions around the glass transition temperature, it was possible to achieve a 13.3% crystallinity, which is around twice higher than that published recently in an investigation related to the influence of the nozzle on the crystallinity, where 7.7% was achieved for an unannealed sample [41]. A grain size between 14.3 and 15.0 µm is a typical value for all PAEKs and it seems to be unaffected by the filler concentration [42].

**Figure 3:**
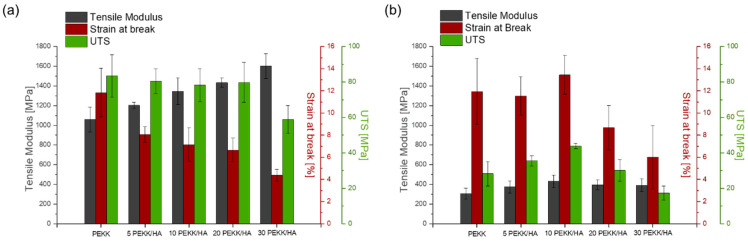
Comparison of the mechanical properties including Tensile modulus, Strain at break, and Ultimate Tensile Stress for different directions of printing: (**a**) XY and (**b**) Z.

The tensile modulus of the specimens printed in the Z-direction was between three and four times lower than that printed in the X-direction (see Figure 3). It is necessary to note that, to stabilize the material during the 3D printing process and prevent warping, it was necessary to preheat the bed to 180 °C, which explains the higher modulus and ultimate tensile strength (UTS), as the specimen was partially crystallized at the bottom layers. Thus, the crystallinity for specimens printed above the glass transition temperature in the XY-direction was higher than that for specimens printed around the glass transition temperature in the Z-direction. The bottom part of the 3DP specimen in the XY-direction turned beige, indicating the formation of crystallites, while for the Z-direction of printing, the gauge-measured section remained amorphous. The Tensile Modulus in the XY-direction rose with a higher content of the filler, with a simultaneous decrease in the strain. As the material was printed under the same conditions, this gradual change was an effect of the rising content of the filler in the composite material, and a similar behaviour was observed in the DMA analysis. Specimens printed in the Z-direction did not follow the same trends. All three parameters of the modulus, UTS, and strain rose with a higher content of the filler up to 10 wt% HA, and after this threshold content, the mechanical performance fell. This can be explained by the fact that the mechanical performance in the Z-direction is largely influenced by the matrix, and a similar observation was confirmed for CF/PEEK [5]. The filler can have a secondary effect on the properties in the Z-direction because 3DP is a non-continuous technique, and when the platform is lowered down, the material is deposited on already solidified material with a weak filler interaction between the layers. The strain at break for the Z printing direction was around 12 MPa up to 10 wt% HA, which was similar to the performance of the matrix in the XY-direction. However, the UTS and tensile modulus were significantly lower, revealing the amorphous character of the specimen printed in the Z-direction but a better, more ductile response to applied stress and the ability to absorb shocks in comparison to the XY-direction. The brittleness of the composites prevailed when the composites were excessively filled with 20 and 30 wt% filler (see Supplementary Figure S2).

The tensile properties in the Z-direction of printing were evaluated after extracting dog-bone-shaped specimens from 120 × 70 × 3 mm thick parts. Each ‘wall’ consisted of three lines that represented the shell of the 3D-printed part. Stress applied during normal use would initially be located on the shell and further distributed to the core of the 3D-printed object. In this investigation, the goal was to measure the properties of the larger object to avoid misleading results obtained from printing individual single dog bones. For small specimens, their overall properties were overestimated due to the continuous heating of lower layers by the heating block during the deposition process. To extract the parts from such a wall, waterjet cutting was used, which is an aggressive method and might lead to a reduction in the overall mechanical properties. Our intention was to determine the material’s suitability for producing implants and orthopaedic and trauma guides in the worst possible orientation during 3D printing. In this configuration, each single deposited layer lay on the previous one, as the shell structure would be in direct contact with the body and be responsible for transferring the initial impact or applied stress.

As the performance of the weakest direction of printing can be considered as the total performance of the part, it was possible to determine that the 10% PEKK/HA composition was the most promising, as it achieved the highest tensile modulus, strain at break, and UTS with values of 432 MPa, 13.4%, and 43.9 MPa, respectively. The performance of the same composition printed in the XY-direction was 1346 MPa, 7.1%, and 78.3 MPa, respectively. These values should be considered as the maximum and minimum boundaries, and the expected properties of the produced parts should lie somewhere in between these two extremes. The final mechanical properties of the part should be evaluated based on how it was oriented in the slicer software, taking into consideration the forces acting on the object during its intended use, whether for implants, orthopaedic guides, or surgical tools.

The selection of the composite with 10 wt% of HA filler aligns with previous work, where, for a different matrix with the same amount of filler, the 10% PEEK/HA composite showed in vivo study results indicating that U-2 OS cells exhibited a flattened morphology and could enhance direct bone apposition in orthopaedic implants [16].

### 3.3. Morphology

**Figure 4:**
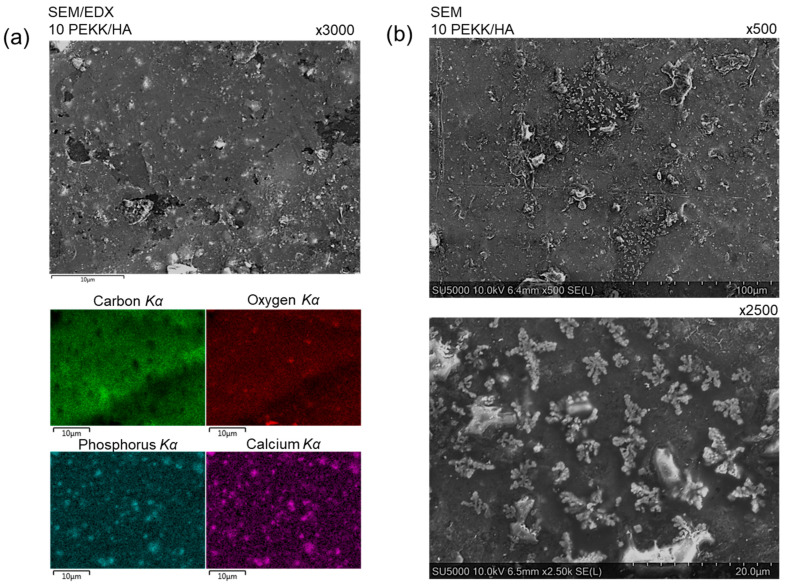
SEM analysis including: (**a**) SEM imaging with EDX elemental mapping for 10 PEEK/HA of carbon, oxygen, phosphorus, and calcium elements, indicating accessibility of filler for cell growing, (**b**) high-resolution SEM imaging for 10 PEKK/HA reviling partially crystalline character of the top surface with magnification ×500 and ×2500.

SEM imaging connected with EDX mapping proved an even distribution of the filler inside the polymer matrix (Figure 4a). In the carbon *Kα* image (green), it is possible to notice empty black spots related to the presence of the HA filler. The phosphorus and calcium *Kα* mapping reveal the distribution of HA as brighter spots of turquoise and pink, respectively. Even though the oxygen element is present in both the HA filler and the PEKK backbone, it is possible to notice more intense spots on the image which overlap with the darker carbon regions, indicating the presence of HA in the polymer matrix as well. This can be noticed across all PEKK/HA compositions, especially when a lower magnification was used for mapping. An elements wt% analysis confirmed not only an equal distribution of the filler, but also successful blending with the desired amount of filler, supporting the TGA analysis (see Supplementary Figure S3). The oxygen, phosphorus, and calcium wt% increased with a rising content of the HA in the composite material, whereas carbon wt% decreased due to the lack of presence of this element in the HA structure.

The SEM images of the top layer of the 3D-printed specimen reveal the semicrystalline character of the matrix with partially crystallized regions. The images were obtained without chemical etching, and the crystals formed at the last layer were observed only after sputtering a thin layer of Au/Pd to provide conductivity, as PEKK and HA are non-conductive materials. Without conductive coating, it was possible to observe beam drift and sample charging. The crystallization process was frozen, and spherulites were not developed due to the slow crystallization kinetics of PEKK. It is possible to observe globules which did not even have time to turn into spherulites (see Figure 4b). The crystallites had a small regional island character surrounded by the amorphous matrix. The diameter of these regions was 80 µm, with an individual crystallite diameter up to 10 µm composed of grains with a diameter around 0.2 µm. Such regional crystallization could be observed over all the studied materials, and the filler content did not affect it (see Supplementary Figure S4).

### 3.4. Crystallinity

**Figure 5:**
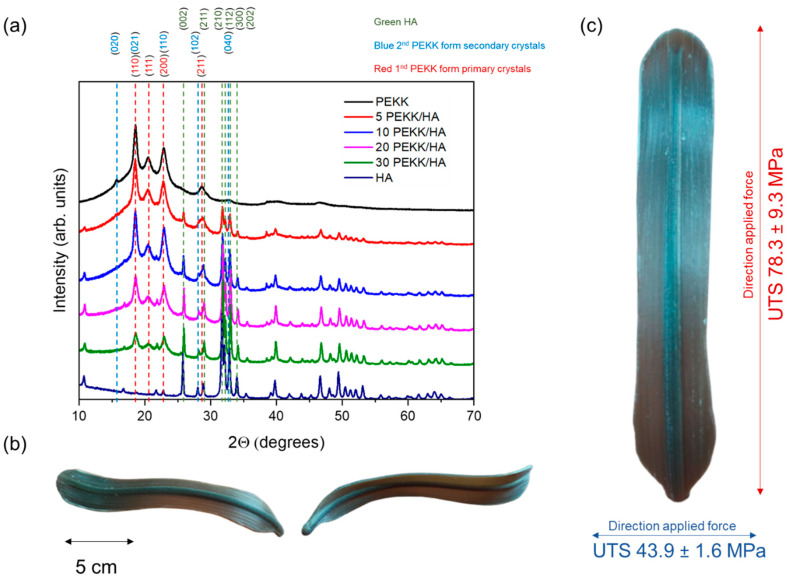
Crystallographic analysis was conducted, including (**a**) XRD diffraction patterns of HA, PEKK, and 5–30 wt% PEKK/HA composites. Additionally, (**b**,**c**) 3D-printed abdominal spatulas used in surgeries to mobilize intra-abdominal structures, with UTS values plotted based on the direction of the applied force and size.

The X-ray diffraction analysis revealed the XRD patterns of the PEKK samples with varying concentrations of HA. As indicated in the diagram, depending on the thermal conditions, PEKK could exhibit two forms of crystal units. Form 1, labelled as “two-chain orthorhombic”, displayed planes at positions of (110) (2*θ* = 18.61°), (111) (2*θ* = 20.49°), (200) (2*θ* = 22.87°), and (211) (2*θ* = 28.67°) and was favoured during melt crystallization (refer to Figure 5a with red numbers). Form 2, represented as “one-chain orthorhombic”, was favoured during cold crystallization with the planes at positions of (020) (2*θ* = 15.71°), (021) (2*θ* = 18.61°), (110) (2*θ* = 21.84°), (102) (2*θ* = 28.15), (120), and (040) (2*θ* = 32.92°) (refer to Figure 5b with blue numbers).

In the composites containing HA, additional peaks at 25.93°, 28.83°, 32.84°, 32.21°, 33.95°, and 35.38° were observed. These peaks corresponded to the (002), (211), (210), (112), (300), and (202) planes of the HA crystal. As the concentration of HA increased, the intensities of these HA peaks, as anticipated, also increased (refer to Figure 5a with green labels).

Based on the intensity of the peaks related to the primary (blue dash lines) and secondary (red dash lines) crystals, it was possible to observe that the predominant crystal variety formed during 3D printing belonged to the more thermodynamically stable primary. These findings aligned with the X-ray analysis of melt-crystallized and cold-crystallized specimens under different conditions for PEKK [18,43]. One of the most common methods giving the ability to improve the mechanical properties of the part and increase the crystallinity is post processing, where the part is kept for a longer period of time under isothermal conditions above the glass transition temperature. However, this might cause the enlargement of the air void due to a shrinking process leading to warping [44]. Additionally, this leads to the formation of less thermodynamically stable secondary crystals. It was shown that temperatures above 310 °C are required to transform secondary crystal into the primary one [14].

This fact is extremely important, because clearly indicates that 3D printing parts crystallized in situ allows only for building the primary crystalline structure, which is directly responsible for mechanical performance and thermal and chemical resistance. This crystallization needs to be formed over a longer period of time and the deposition of the new layer should be performed on amorphous material to avoid semicrystalline material to form the crystals, as they have a higher thermal stability and lead to a poor interlayer connection, as was noted for fast-crystallization PEEK [45]. For this reason, the deposition process should occur on the previously deposited material while it is in the amorphous state to prevent the melting of stable crystallites, which have a thermal resistance of up to 260 °C. The amorphous phase, which can withstand temperatures up to the glass transition point of 160 °C for PEKK, allows the polymer chains to effectively bond with the newly deposited layer and form interconnections throughout the entire 3D-printed part. Then, by keeping the specimen above the glass transition temperature, slowly crystalizing PEKK can form crystals over a longer period, as additive manufacturing is a relatively slow method, giving the required and tuneable mechanical performance and thermal and chemical resistance.

## 4. Conclusions

To the best of the author’s knowledge, this is the first report on the extrusion of PEKK/HA composite filaments used in 3D printing with a custom, cost-effective modified printer. For the first time, the tensile performance in the Z- and XY-directions of the printing process was determined and thoroughly explained, taking into account a key material parameter: the kinetics of crystallization. It was determined that only the in situ crystallized parts could be characterized as being rich in the primary form of crystallites, while post-processing mostly generated a less thermodynamically stable secondary form of crystallites.

PEKK and composites with different HA loadings were investigated to evaluate their usefulness as promising materials for making implants, orthopaedic guides, and trauma devices. Tensile analysis revealed the orthotropic character of the 3D-printed composites, and specimens exhibited distinct characteristics in the XY- and Z-printing directions.

The PEKK 6002 composites provided a better thermal stability during autoclave sterilization, typically conducted at around 120 °C, with a glass transition temperature around 160 °C compared to PEEK, which has a *T_g_* of 143 °C. Interlayer bonding, essential for developing materials with a more orthotropic performance under applied loads and ensuring the mechanical integration of implants, was significantly better for the PEKK/HA composites due to the reduced crystallization kinetics of the polymeric matrix. PEEK printed under the same conditions was characterized by weak interlayer connections, resulting in delamination, whereas for 10 wt% PEKK/HA, it was possible to achieve 43.9 MPa. PEKK, due to its amorphous character, possesses a superior wear resistance and higher strain, as was demonstrated in this work. The main disadvantages of PEKK/HA were connected to the reduced crystallinity and more amorphous nature of the composites compared to other materials with faster crystallization kinetics, such as PEKK 7000, PEKK 8000, and PEEK. These disadvantages include a lower modulus of elasticity and limited chemical resistance. However, materials with slower crystallization kinetics allow for the tuning of final properties through printing conditions, which is not achievable with PEEK due to its faster crystallization kinetics.

The study of crystallization kinetics showed that the filler acted as a nucleating agent only at low concentrations, and an increase in the crystallization rate was observed only for 5 wt% HA.

SEM imaging presented a frozen type of crystalline structure at the top of the 3D-printed specimens for the polymer matrix with relatively slow crystallization kinetics. Most of the matrix was in an amorphous state, providing ductility and impact resistance. This, together with EDX mapping that showed an equal distribution of HA particles across the surface of the 3D-printed samples, allowed us to claim that these materials are promising for use in composite materials, particularly for orthopaedic surgical guides, such as abdominal spatulas (see Figure 5b). However, when it comes to implants, it is necessary to evaluate the mechanical properties of the desired shape to optimize the printing orientation against the applied stress during usage, as the body will have an orthotropic character.

Unambiguously, the most promising composition was 10% PEKK/HA, as this specimen exhibited the highest mechanical performance in the Z-direction of printing, which was identified as the weakest direction, determining the performance of the whole body (see Figure 5c). For HA contents of 10 wt% and above, it was possible to observe slower crystallization kinetics. The filler’s contribution to the mechanical properties, measured in the Z-direction of printing, had a secondary effect, and the obtained properties, measured with stress applied perpendicular to the layer, primarily reflected the interlayer matrix-to-matrix connection for filler concentrations between 0 and 10 wt% HA. At larger amounts, 20 and 30 wt% HA, due to the extensive loading of the material with mineral type filler, the load was not efficiently transferred and dispersed by the matrix, leading to a significant reduction in strain.

## Figures and Tables

**Table 1:** Overview of mechanical and microstructural characteristics for 3D-printed specimens in the Z- and XY-directions.

Z Printing Direction	PEKK	5 PEKK/HA	10 PEKK/HA	20 PEKK/HA	30 PEKK/HA
Modulus [MPa]	304 ± 55	374 ± 61	432 ± 63	396 ± 50	392 ± 66
UTS [MPa]	28.2 ± 7.0	35.5 ± 2.9	43.9 ± 1.6	30.0 ± 6.1	17.4 ± 3.9
Strain [%]	11.9 ± 3.0	11.5 ± 1.7	13.4 ± 1.7	8.7 ± 1.9	6.0 ± 2.9
**XY Printing Direction**					
Modulus [MPa]	1059 ± 125	1202 ± 33	1346 ± 136	1434 ± 47	1602 ± 127
UTS [MPa]	83.4 ± 12.0	80.5 ± 6.9	78.3 ± 9.3	79.8 ± 11.2	58.9 ± 7.9
Strain [%]	11.8 ± 2.2	8.1 ± 0.7	7.1 ± 1.5	6.6 ± 1.1	4.4 ± 0.6
*X_c_^XRD^* [%]	13.1	14.4	12.5	10.6	8.3
Grain Size [nm]	14.8	14.7	14.3	15.0	14.7

## Data Availability

The original contributions presented in the study are included in the article/Supplementary Materials, further inquiries can be directed to the corresponding authors.

## Supplementary Materials

The following supporting information can be downloaded at: https://www.mdpi.com/article/10.3390/ma17133161/s1, Figure S1: DSC isothermal study of PEKK materials with HA filler content between 0–30 wt%. Crystallization peak time depends on the temperature during isothermal conditions; Figure S2: Comparison of the tensile properties for different direction of printing XY left and Z right for selected specimens; Figure S3: SEM imaging with EDX elemental mapping Carbon, Oxygen, Phosphorus and Calcium elements of PEKK materials with HA filler content between 0–30 wt%; Figure S4: High resolution SEM imaging for PEKK materials with HA filler content between 0–30 wt% of top surface with magnification between ×500 and ×20,000.
