# Supplementary material for: Advanced 3D Printing of Polyetherketoneketone Hydroxyapatite Composites via Fused Filament Fabrication with Increased Interlayer Connection
Source: Materials (Basel). 2024 Jun 27;17(13):3161. doi: 10.3390/ma17133161 (PMC11242051; doi:10.3390/ma17133161)
Supplement: Supplementary file 1 [file materials-17-03161-s001.zip › materials-3050846-supplementary.pdf]

Supplementary Information

Advanced 3D Printing of Polyetherketoneketone Hydroxyapatite Composites via Fused Filament Fabrication with Increased Interlayer Connection

Krzysztof Rodzeń [1,*], Eiméar O'Donnell [1], Frances Hasson [1], Alistair McIlhagger [1], Brian J. Meenan [1], Jawad Ullah [1], Beata Strachota [2], Adam Strachota [2], Sean Duffy [1] and Adrian Boyd [1,*]

[1] School of Engineering, Ulster University, York St, Belfast BT15 1ED, UK; odonnell-e19@ulster.ac.uk (E.O.); hasson-f3@ulster.ac.uk (F.H.); a.mcilhagger@ulster.ac.uk (A.M.); bj.meenan@ulster.ac.uk (B.J.M.); j.ullah@ulster.ac.uk (J.U.); duffy-s36@ulster.ac.uk (S.D.)

[2] Institute of Macromolecular Chemistry v.v.i., Academy of Sciences of the Czech Republic, Heyrovskeho nam. 2, 162 00 Praha, Czech Republic; beata@imc.cas.cz (B.S.); strachota@imc.cas.cz (A.S.)

* Correspondence: kp.rodzen@ulster.ac.uk (K.R.); ar.boyd@ulster.ac.uk (A.B.)

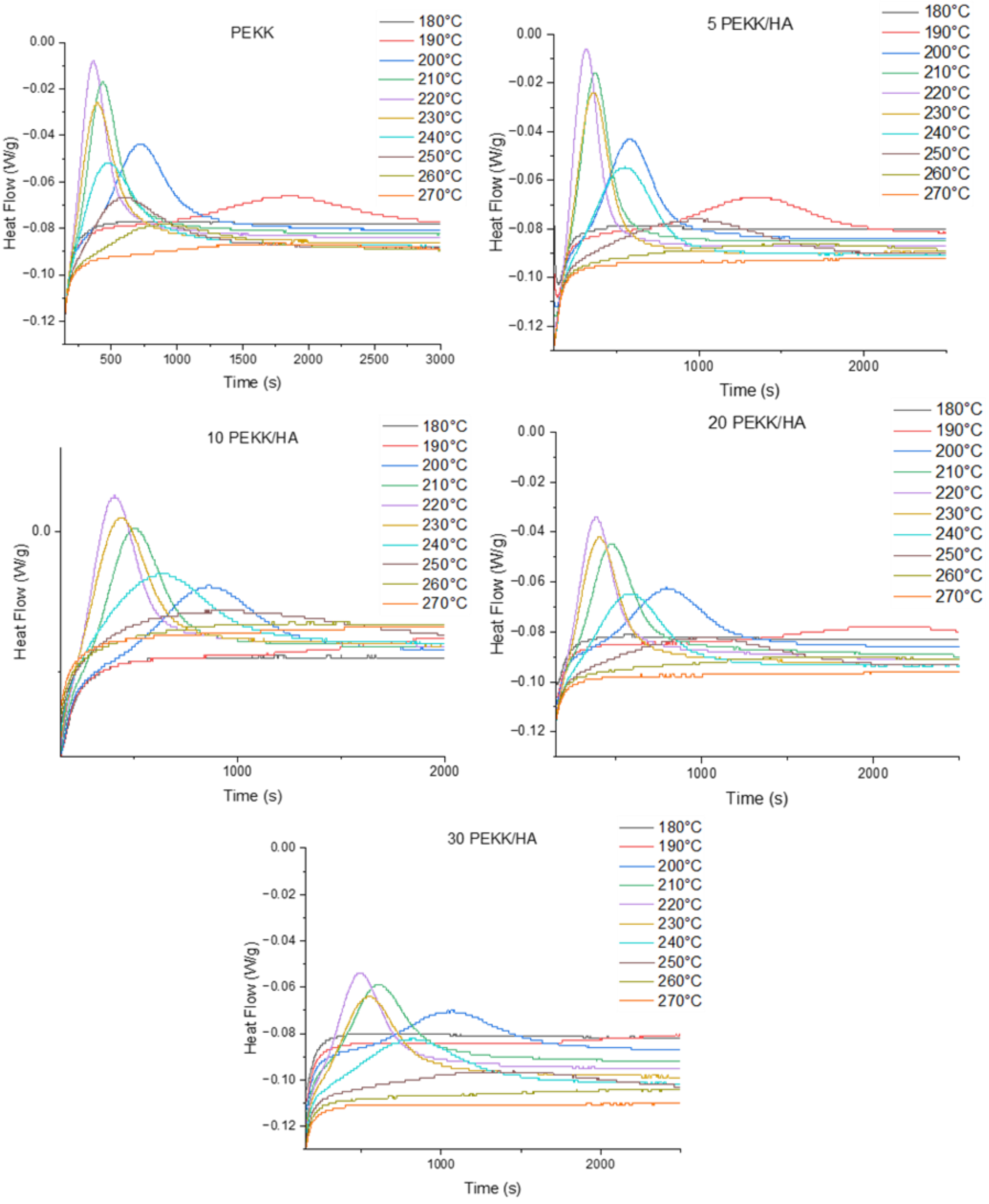

Supplementary Figure S1. DSC isothermal study of PEKK materials with HA filler content between 0-30 wt%. Crystallization peak time depends on the temperature during isothermal conditions.

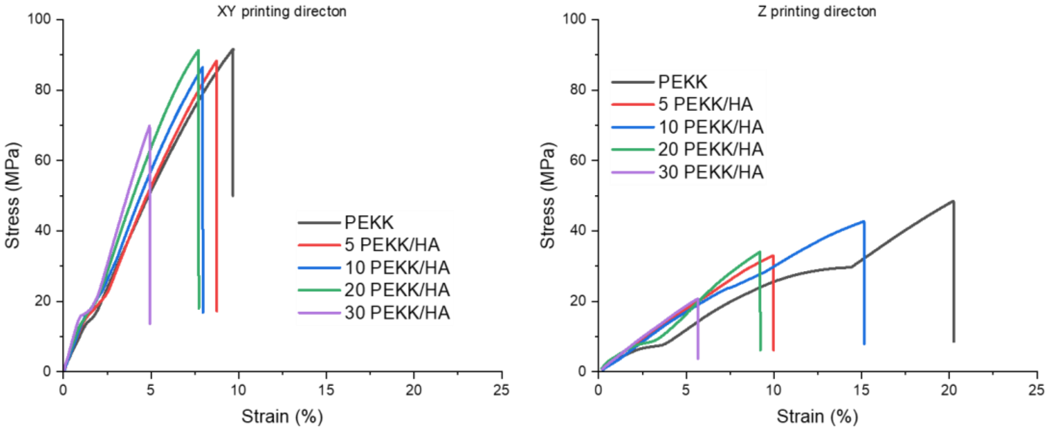

Supplementary Figure S2. Comparison of the tensile properties for different direction of printing XY left and Z right for selected specimens.

HAp Content [wt%]	0		5		10		20		30	
	**Element**	**Wt%**	**Element**	**Wt%**	**Element**	**Wt%**	**Element**	**Wt%**	**Element**	**Wt%**
	C	81.3	C	74.71	C	72.66	C	66.22	C	60.52
	O	18.68	O	22.77	O	23.02	O	23.95	O	24.85
	P	0	P	0	P	0	P	2.12	P	3.56
	Ca	0.02	Ca	2.52	Ca	4.32	Ca	7.71	Ca	11.06
	**Total**	100	**Total**	100	**Total**	100	**Total**	100	**Total**	100

X 1000 Mag

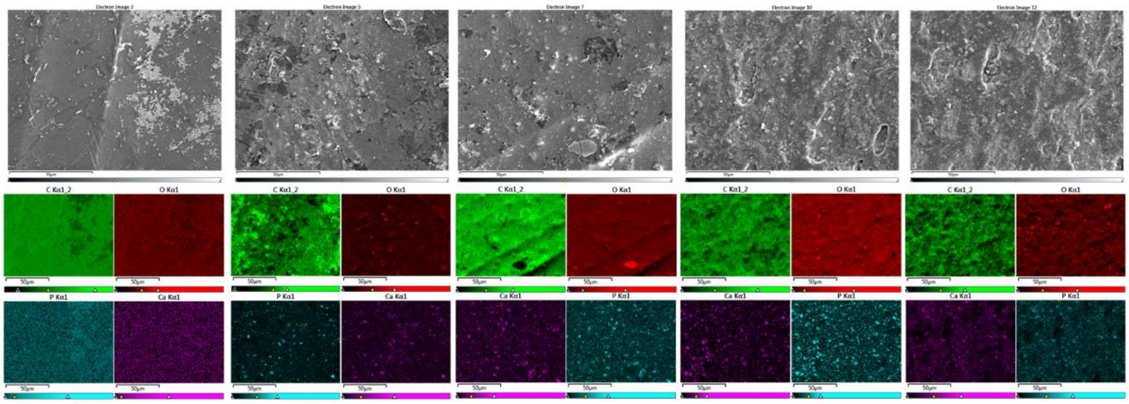

Supplementary Figure S3. SEM imaging with EDX elemental mapping Carbon, Oxygen, Phosphorus and Calcium elements of PEKK materials with HA filler content between 0-30 wt%.

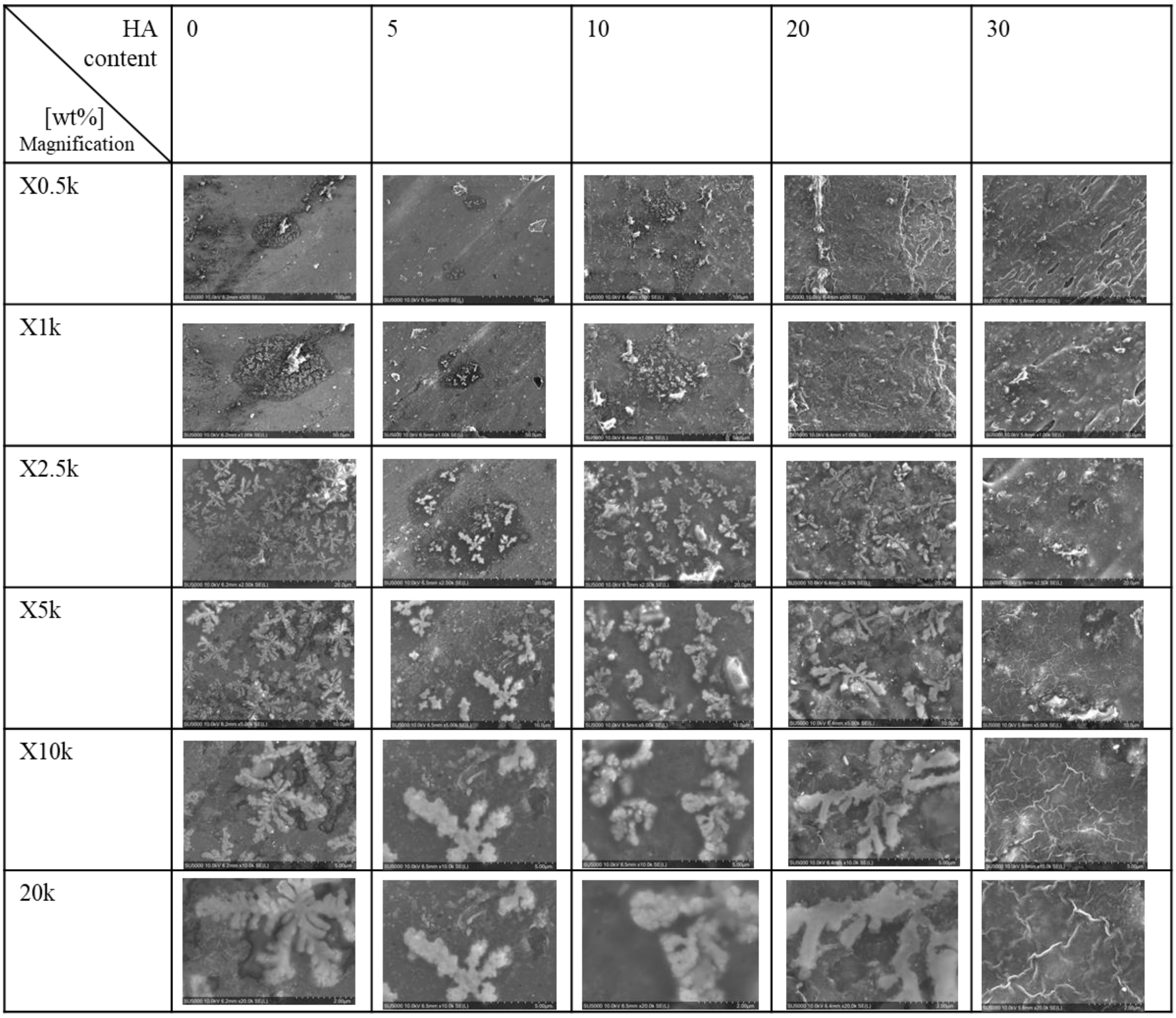

Supplementary Figure S4. High resolution SEM imaging for PEKK materials with HA filler content between 0-30 wt% of top surface with magnification between x500 and x20 000.
